# Advanced Characterization and Rejuvenation of End‐Of‐Life Lithium‐Ion Anodes: Toward the Development of a Green Upcycling Route

**DOI:** 10.1002/smll.202512626

**Published:** 2026-01-20

**Authors:** Luke Sweeney, Alexander T. Sargent, Yuhang Dai, Shangwei Zhou, Jianuo Chen, Francesco Iacoviello, Rhodri Jervis, Phoebe K. Allan, Peter R. Slater, Charles Monroe, Paul A. Anderson, Paul R. Shearing, Wenjia Du

**Affiliations:** ^1^ School of Chemistry University of Birmingham Birmingham UK; ^2^ The Faraday Institution Quad One Harwell Science and Innovation Campus Didcot UK; ^3^ Department of Engineering Science University of Oxford Oxford UK; ^4^ Electrochemical Innovation Lab Department of Chemical Engineering University College London London UK; ^5^ The ZERO Institute University of Oxford Oxford UK; ^6^ Oxford Martin School University of Oxford Oxford UK

**Keywords:** 3D microstructure, graphite electrode, recycling, regeneration, X‐ray tomography

## Abstract

The limited duty cycles of EV batteries necessitate robust end‐of‐life strategies to prevent landfilling and enable responsible resource management. Recycling remains the ultimate fate for battery waste, yet current hydrometallurgical practices rely on often lengthy and energy‐intensive methods, which provide a major incentive for the development of cost‐effective, shorter‐loop regeneration routes to recycle end‐of‐life electrodes. This proof‐of‐concept study investigates the efficacy of deionized (DI) water and ascorbic acid (AA) in recovering entire spent anode systems from retired EV batteries without delamination and re‐manufacture. Multi‐modal characterization techniques were used to evaluate electrochemical, physicochemical, and morphological changes before and after treatment. High‐resolution X‐ray tomography and image‐based simulations were used to quantify the microstructural metrics of pretreated and rejuvenated anodes. 3D visualizations of graphite and pore phases revealed insights into recycling mechanisms and rejuvenation effectiveness. Results demonstrate that DI water effectively removed surface impurities on graphite, significantly enhancing regeneration performance, with stable discharge capacities of ∼2.65 mAh/cm^2^ over 20 cycles at 0.1 C, which exceeded both unused (2.36 mAh/cm^2^) and end‐of‐life (0.56 mAh/cm^2^) anodes. The study demonstrates that green chemistries can offer a sustainable alternative to hydrometallurgy and highlights the vital role of X‐ray imaging in advancing circular battery technologies.

## Graphical abstract

Green solvents (DI water or ascorbic acid) enable the regeneration of end‐of‐vehicle‐life battery anodes without delamination and re‐manufacture. Multimodal characterizations reveal several graphite microstructures and crystallography, and restored cycling performance. We demonstrate a sustainable route for anode rejuvenation and circular battery materials recovery.

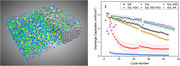

## 1 Introduction

The rapidly increasing number of electric vehicles (EVs) presents a significant waste‐management challenge for society at the end‐of‐life (EoL) [1]. Recycling and rejuvenation of EoL components and materials in retired Li‐ion batteries (LIBs) is thus critical for developing a sustainable future [2, 3]. Although legislation (e.g., UK Waste Batteries and Accumulators Regulations [4]) has been rolled out by many authorities mandating an ever‐increasing percentage of recycled materials in new batteries, outdated and rudimentary recycling technologies with low recovery rates, and inadequate infrastructure remain significant bottlenecks.

Historically, battery recycling has been primarily focused on cathode recovery [5, 6]. Battery cathodes containing valuable metals such as Li, Co, Ni, and Mn have a higher economic value than battery anodes [7], making cathode recycling a more lucrative endeavor. Consequently, there is limited literature on the recycling, regeneration, and re‐utilization of graphite materials from spent LIBs at the end of vehicle life.

Recently, substantial attention is being directed toward battery anode recycling [8]. Increasing demand from the automotive industry has led to a surge in demand for graphite, with more than 8 Mt required for clean energy by 2030 according to the IEA [9]. Although synthetic graphite has been widely used to meet the needs of battery manufacturing, producing synthetic graphite is extremely energy‐intensive and expensive, often requiring a heating environment above 2500°C [10]. Recycling secondary graphite from e‐waste and vehicle batteries is in principle a more sustainable route than producing and refining the primary graphite, helping to minimize ore extraction, mitigate the risk of battery waste‐management issues, and stabilize the supply chain for energy security. Furthermore, the recovered graphite can be used as a precursor to synthesize advanced chemistries for many other purposes beyond battery applications such as graphene [11], catalysts, and capacitors. Modification of regenerated graphite through doping [12], coating [13], and compositing [14] techniques provides superior cycling stability, demonstrating the added value of anode recycling, albeit adding to processing costs.

In general, battery recycling employs shredding techniques to produce a mixed ‘black mass’ powder [15, 16], which is further refined through energy intensive pyrometallurgical or ‘long‐loop’ hydrometallurgical processes [17]. This methodology, however, fails to consider the manufactured value of the object being recycled. For the case of an anode found in a mass‐produced cell, this component is the product of 1000's of hours of testing and optimization, often manufactured to a specification unobtainable within research labs. Thus, by shredding or delaminating a commercially made electrode, the product becomes greatly devalued.

It is also crucial to develop facile techniques to recycle LIBs locally to minimize environmental and social impacts. Washing techniques using green solvents such as deionized (DI) water and organic acids (such as citric and ascorbic acids [18, 19]) have been considered as a promising solution to remove contaminants and assist anode recycling. Cost‐effective, green reagents avoid the unnecessary need for harsh chemicals, such as strong acids, making it safer for recycling operations, minimizing hazardous waste, helping to conserve natural resources and, importantly, minimizing chemical damage to the electrode assembly and active material.

Although the washing approach [20] has been widely used to generate recovered products with a higher purity than shredding, the underlying mechanisms of purification on washing remain poorly understood. There are limited research articles using green solvents to regenerate the graphite [19, 21]; however, waste electrodes are usually recycled, regenerated, and wet‐manufactured (*via* slurry casting) after a step or steps to separate the active materials and current collectors. Unfortunately, from a life cycle assessment (LCA) point of view [22], the entire regeneration procedure, from EoL to the reuse of waste materials in ‘new’ cells, is long and costly, often including heat treatment [23] to repair the defective graphite materials, and produces low yields of reusable material. This provides a major incentive for us to develop shorter‐loop regeneration routes.

Characterization has been undertaken to understand the interaction between rejuvenation agents, the graphite particles, and aging‐induced impurities via scanning electron microscopy (SEM) and transmission electron microscopy (TEM) [8], but the information obtained is relatively limited, and the dominant underlying factors determining the rejuvenation quality remain far from clear. As a result, researchers often rely on ‘trial and error’ to explore recycling processes and performance on regeneration.

Direct observation of recovered structures offers a pathway to bridge the missing links between washing techniques and electrochemical characterization by quantifying the effectiveness of the washing agents. X‐ray tomography, as a non‐destructive tool, has been successfully employed to characterize internal structure and morphology in 3D, significantly accelerating fundamental research across various themes in materials science [24, 25, 26, 27], such as batteries and fuel cells [28, 29]. Owing to its large window of spatial resolution (which spans tens of millimeters to tens of nanometers), X‐ray tomography has been extensively used for quality assurance and to understand battery degradation in early life across multiple length scales, covering particles to electrodes and entire devices [30, 31]. Additionally, it offers possibilities to extend investigations to 4D (3D plus time), which correlates structural dynamics to the performance and lifetime [32, 33, 34].

In this study, EoL anodes, before and after washing treatments using green solvents, were characterized for the first time by high‐resolution X‐ray tomography, without being removed from the metal current collector. The effectiveness of the washing treatment was morphologically and chemically characterized by multiple tools including tomography to correlate the performance after regeneration with 3D microstructures. In particular, the porosity and connectivity of the recovered graphite in situ on the electrode were visualized and quantified to help understand how washing can remove the residues and impurities (such as metal oxides formed on the graphite surface) within EoL anodes and enable the functionality of the spent graphite to be restored.

## 2 Experimental Methods

### 2.1 Samples

The LIBs used in this study were large‐format pouch cells recovered from an EoL Nissan Leaf vehicle (gen. 1). The cells were torn down to harvest the electrode layers in an inert‐atmosphere glovebox. All samples were exposed to air for at least one month prior to use. This was carried out to simulate the conditions of real‐life recycling operations where after an initial passivation step materials are generally exposed to air for some time. A summary table of the samples investigated in this paper is listed in Table 1 showing their life history and information. The EoL electrodes were maintained as double‐sided during the recycling and characterization. Single‐sided graphite electrodes were used for electrochemical testing.

**TABLE 1:** Details of the seven types of graphite electrode used in this study.

Name	Origin	History and description
1. Quality assurance reject (QA)	Gen. 1	Manufactured and formation cycled. Rejected by the QA process, after which it was discharged to 0 V.
2. End‐of‐life (EoL)		Passed quality control testing and was used in a Nissan Leaf vehicle. After 40 000 miles and 12 years on the road, the EV batteries were retired and dismantled at the Universities of Newcastle and Birmingham.
3. End‐of‐life 500 (EoL 500)		EoL pouch cell (history above). The cell was cycled an additional 500 times between the voltages of 2.5–4.4 V using the GCPL program with no voltage hold step.
4. End‐of‐life water‐washed (EoL H_2_O)		EoL pouch cell (history above). The cell was dismantled and washed with DI water.
5. End‐of‐life 500 water‐washed (EoL 500 H_2_O)		EoL pouch cell (history above). The cell was cycled an additional 500 times before being dismantled and washed with DI water.
6. End‐of‐life ascorbic acid (EoL AA)		EoL pouch cell (history above). The cell was dismantled and washed with ascorbic acid.
7. Unwetted	Gen. 2	Dry electrode manufactured for a gen. 2 Nissan Leaf pouch cell. This electrode reel was never used within a battery.

### 2.2 Regeneration via Green Washing Agents

Ascorbic acid (AA) powder (Sigma–Aldrich) was dissolved in deionized water to make a 1.5 m solution. In this study, AA and neat DI‐water were used as washing solutions. Pretreated samples were gently put into the liquid medium (either AA or DI‐water) using tweezers to ensure the electrode sank within the liquid medium (the volume of washing solution was a minimum of 10 mL). Some effervescence occurred once the graphite surface of the electrode contacted the acidic solution. Upon sinking, effervescence continued for around 10 min. When the electrodes were washed in DI‐water, effervescence occurred but at a much slower rate compared to the AA solution. After AA washing, the treated electrodes were briefly washed in DI‐water three times, and all washed samples were dried in a drying oven set to 60°C.

### 2.3 Morphological Characterizations via X‐Ray Tomography

The size of double‐sided electrode was reduced to a ‘tab’ of ca. 0.5 mm × 0.4 mm using a micro‐milling laser technique (A Series/Compact Laser Micromachining System, Oxford Lasers, UK). A high‐resolution µ‐CT instrument (Zeiss Xradia 620 Versa, Carl Zeiss Inc.) was used to collect tomography of graphite samples. A 20 X lens was used, and a binning of 1 was set on the 2048 × 2048 pixels CCD detector to achieve pixel sizes of 419 nm with field of views (FOV) of ca. 860 µm × 860 µm. A polychromatic cone‐beam source using a tungsten target with the voltage set at 100 kV was used to generate a total power of 14 W. A total of 1601 projections were obtained with an exposure time of 15 s. 2D projections were reconstructed using Zeiss software (XMReconstructor, Carl Zeiss Inc.). The center of rotation was calculated automatically, and a beam hardening correction was applied during both reconstruction protocols. Additionally, an advanced reconstruction algorithm (MARS) was used to enhance the quality of scans by minimizing the beam hardening [35]. The reconstructed images with and without MARS are demonstrated in Figure S1.

Visualization and analysis of the reconstructed datasets were carried out using Dragonfly 2024 software (Comet Technologies Canada Inc.). A Contrast Limited Adaptive Histogram Equalization (CLAHE) filter was applied to improve the contrast of images. The images with and without CLAHE filter are presented in Figure S2. A deep‐learning (U‐net) model was generated and trained to identify and classify all features within the anode, including current collectors, graphite particles, carbon‐binder domain (CBD), and exterior based on the grayscale and feature's shape and orientation. The carbon‐binder and porosity were segmented as one phase due to similar attenuation and contrast. Several 2D ground‐truth frames at multiple local regions with characteristic features were manually labeled to train this model. The profile of the training procedure consisting of the epochs and the loss function is shown in Figure S3. The trained model was then applied to automatically segment the entire volume data for each sample. The Pore Network Model (PNM) module [36] in Dragonfly was also employed to explore the porosity and connectivity in porous electrodes. Taufactor [37] was used to simulate the Li^+^ flux, which reflected the ion transportation and generate tortuosity and percolation numbers.

### 2.4 Physicochemical Characterization

SEM images and energy dispersive spectroscopy (EDS) analysis of pretreated and regenerated samples were taken using a Zeiss EVO 10 and a Hitachi 4000plus benchtop with an AztecOne EDX analyzer.

Powder X‐ray diffraction (XRD) patterns of electrodes were collected before (Figure S4) and after (Figure S5) washing using a Malvern Panalytical, Empyrean diffractometer (Cu source 𝜆 = 0.154 nm) with the electrodes placed on a low background holder. All XRD data were fitted and analyzed in the Origin Pro software using a bigaussian peak fitting function.

Raman spectra for the QA and EoL graphite electrodes (Figure S6) were acquired using a Horiba LabRam Aramis Raman spectrometer, using a 532 nm laser with at 0.050 mW power at 10% power setting, using 1800 L/mm grating, where spectra were acquired using 1 accumulation for 30 s. For each sample, 9 spectra were acquired over a 2 mm × 2 mm grid (spaced 1 mm apart).

Mercury intrusion (AutoPore IV 9500) was also performed to measure the pore size of the associated electrodes as shown in Figure S7.

### 2.5 Electrode Preparation, Cell Assembly, and Electrochemical Characterization

All double‐sided electrodes were secured to a large hotplate, which was set to 60°C. A soft abrasive sponge soaked in warm N‐methylpyrrolidone (NMP) (or DI‐water in the case of the unwetted electrode) was used to gently rub the graphite coating off one side of the electrode (to facilitate coin‐cell testing). Once the graphite was removed, exposing the copper side, the electrode was left on the hotplate at 60°C until dry.

The now single‐sided graphite electrodes were cut to a diameter of 12 mm and dried overnight in a vacuum oven at 110°C. After drying, coin cells (CR 2032) were assembled within an argon‐filled glove box (MBraun, O_2_ and H_2_O content below 1 ppm) using the graphite electrode, separator (Whatman GF/C glass microfiber), 50:50 EC:DMC electrolyte (160 𝜇L) 1 M LiPF_6_, and lithium metal foil with a diameter of 15 mm.

Electrochemical testing of graphite materials was carried out using the CR2032 coin cells between the voltage range of 0.005–1.5 V. All coin cells were formation cycled via galvanostatic cycling with potential limitation (GCPL) at a rate of C/40 (9.3 mAh/g) for three cycles then C/10 (37.2 mAh/g) for 40 cycles using a BioLogic BSC‐800 series battery cycler. Electrochemical impedance spectroscopy (EIS) measurements and the transmission line model (TLM) were performed to extract the resistance and capacitance values of each physical process within the electrode.

## 3 Results and Discussion

### 3.1 Benchmark of Anodes Before Washing

The electrochemical, physicochemical and morphological measurements were performed on Leaf gen. 1 anodes after formation, end‐of‐life and aggressive aging simulating a non‐optimized second‐life application (EoL 500). An unwetted anode for a Leaf gen. 2 cell was included in the study purely for comparison purposes.

Two dominant peaks can be observed from the XRD pattern, with the graphite (002) peak at ∼ 26^>°^ 2𝜃 and the copper (111) peak at ∼ 44^°^ 2𝜃 (Figure S4). The results show minimal difference in the graphite crystal structure from pristine state to EoL 500, suggesting a degraded graphite structure is not the primary cause of capacity fade.

The Raman spectra acquired for the graphite anodes contain the G peak (1580 cm^−1^) and 2D peak (2700 cm^−1^), characteristic of sp^2^‐bonded graphite, and the D peak (1350 cm^−1^) which requires defected 6‐membered sp^2^‐bonded carbon rings for activation (see Figure S6). The intensity ratio of the G and D peaks, I(D)/I(G), is indicative of the degree of disorder in graphitic flakes with lateral dimension greater than ∼20 Å [38], with lower I(D)/I(G) corresponding to fewer defects. The median I(D)/I(G) ratios for the QA reject and EoL samples are 0.93 and 0.65 respectively, suggesting increased degree of defection in the EoL sample. It is also noticed that the broad peak at c. 600 cm^−1^, which is consistently present in the EoL sample over the 2 × 2 mm^2^ region characterized, is consistent with a peak attributed to salt residue in EoL reclaimed graphite in Ref [39].

Figure S8 shows that the average weight of anode increased with lifespan. It is believed that the battery degradation [40, 41], including solid–electrolyte interphase (SEI) formation, manganese migration, and copper oxidation, etc., has strong correlation with the observed mass increase.

**FIGURE 1:**
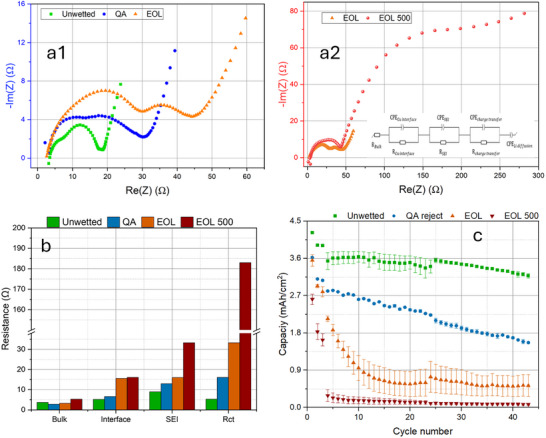
The EIS and cycling performance for pristine, end‐of‐life, and second‐life samples, before regeneration. (a) Nyquist plots of the impedance results after the formation are presented for unwetted, QA reject, EoL, and EoL 500. Equal circuit (inserted) using transmission line model (TLM) was used for fitting the associated EIS data; (b) comparison of the bulk, interface, SEI, and charge transfer resistance extracted from (a); (c) the discharge capacity as a function of cycle number for the associated samples, with the error bars representing the standard deviation.

Figure 1a displays the EIS collected after formation. The spectra were fitted using the TLM model, (see Figure 1a2). The sample after second‐life (EoL 500) had the highest resistance of all, attributed to its extremely high charge transfer resistance. The unwetted anode had the lowest resistance, followed by the QA reject and EoL ones.

In Figure 1b, there is a gradual increase in *R*_bulk_ as a function of aging from pristine state (QA reject, 2.79 Ω) to end‐of‐life (3.27 Ω) to second‐life state (EoL 500, 5.29 Ω). *R*_bulk_ can be used as an aging parameter to indicate cell degradation [42]. The increase in *R*_bulk_ is attributed to a combination of factors, including SEI formation, electrolyte degradation, decomposition, and loss of contact between active materials. *R*_SEI_ also exhibited a clear age dependency with an increase from 12.95 to 33.32 Ω, corresponding to pristine and second‐life cases. *R*_𝑐𝑡_ showed the largest change (from 12.67 to 16.67 to 183.01 Ω), which suggests that lithium diffusion through (de)intercalation should be the primary reason for the degraded cycling performance (discussed below). A large shift within the frequency range is observed between the EoL and EoL 500 with the *R*_𝑐𝑡_ being shifted to the mHz range indicating extremely slow kinetics, which is the key factor that governs the aging process.

Figure 1c shows the cycling performance of all the anodes. The data showed a significant fade in capacity for both the EoL 500 and EoL anodes after the 5th cycle, especially the EoL 500 whose capacity dropped from 2.65 to 0.28 mAh/cm^2^ after just 3 cycles. The unwetted anode demonstrated exemplary cycling performance (the best Coulombic efficiency of all), which was also supported by EIS data. Its capacity decreased from 4.25 to 3.6 mAh/cm^2^ due to SEI formation, then was maintained around 3.5 mAh/cm^2^ until the 25th cycle. The QA reject anode presented a linear trend in terms of capacity fade after the formation cycle.

Data from multiple anodes measured through destructive mercury intrusion measurements, including pore density, porosity, and tortuosity, are summarized in Table 2 and will be compared to those obtained by X‐ray tomography (discussedbelow).

**TABLE 2:** Data extracted from mercury intrusion porosimetry.

Sample	Pore density (g/mL)	Porosity (%)	Tortuosity
QA	N/A	44.69	3.58
EoL	0.13	33.84	3.78
EoL 500	0.20	38.00	3.89
Unwetted	N/A	38.11	3.85

**FIGURE 2:**
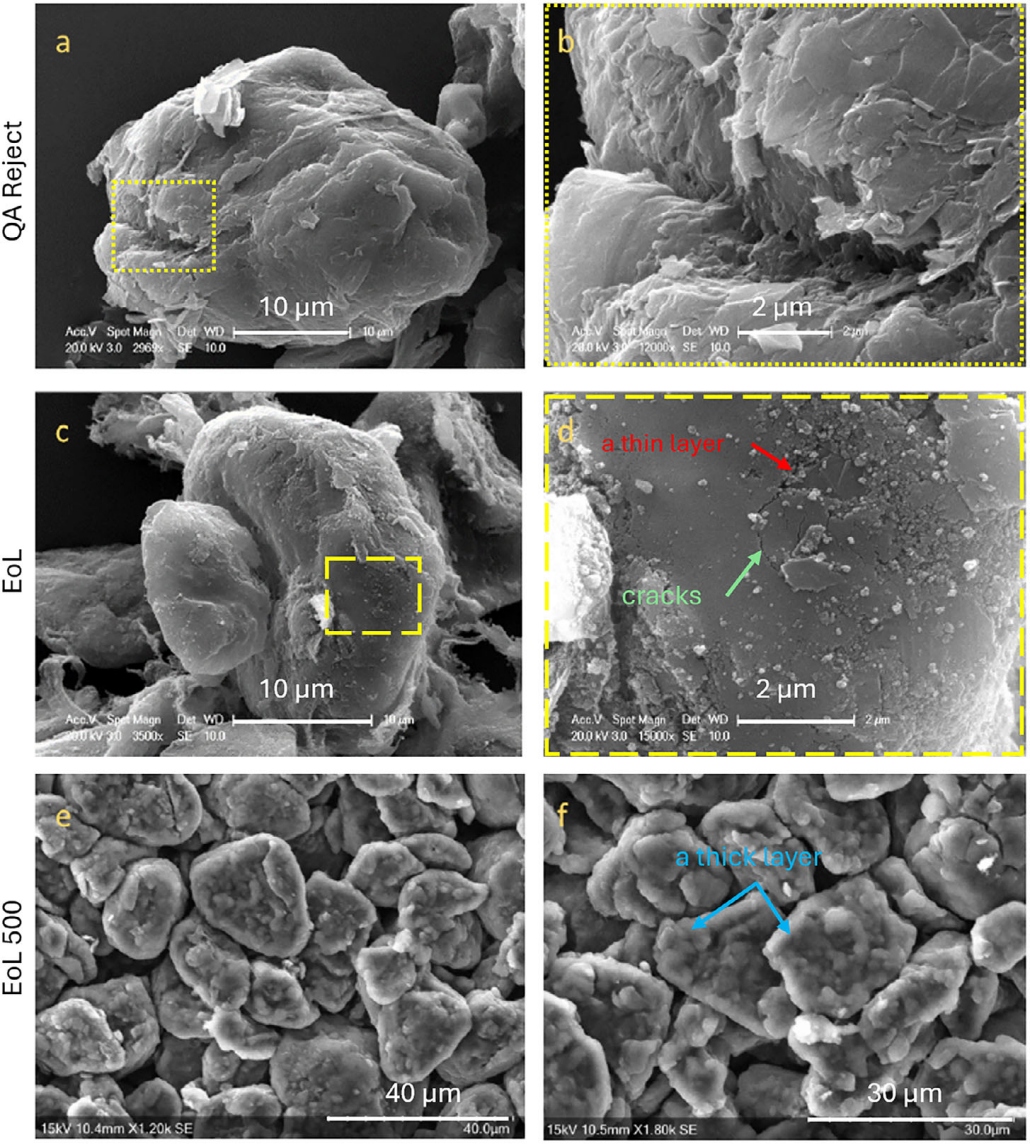
SEM images showing 2D microstructures after various lifetimes, including (a,b) QA reject (pristine state); (c,d) cycled anode at end‐of‐life; (e,f) second‐life anode after EoL. Note: EoL 500 images were taken on a Hitachi bench top microscopy, whilst the EoL and QA samples were taken on Zeiss Evo 10 microscopy.

SEM images of the graphite in pristine, end‐of‐life, and second‐life states are shown in Figure 2. A typical image of a specialized EV battery grade graphite particle is presented to illustrate the QA anode material (Figure 2a), whilst a zoomed‐in image in Figure 2b highlights the polycrystalline structure of the graphite.

In contrast, images of end‐of‐life (EoL) graphite are shown in Figure 2c,d, where the crystalline features appear less prominent compared to the original state. In Figure 2d, the original graphite surface appears to be covered by a cycle‐induced surface layer with a small 2 µm crack. The presence of a surface coating is consistent with the aforementioned increased mass (Figure S8), suggesting the buildup of organics and carbonates on the graphite surface, impeding lithium movement during the charge transfer process.

The second‐life (EoL 500) anode is presented in Figure 2e,f for comparison with the end‐of‐life and pristine cases. Clearly, surface contamination worsened with aging, as large clusters of amorphous materials were observed coating all the graphite particles. The thickness of the coating layers appears uneven, indicating inhomogeneous degradation in localized regions.

### 3.2 Spent Anodes After Rejuvenation

Before structural analysis, the washing effect was initially assessed by measuring the electrode mass change. Figure S9 compares the masses of different electrodes before and after washing, showing a decrease in electrode mass of between 1.5 and 2 mg following the washing treatments. The results suggest that water and AA solution help dissolve residues and impurities agglomerated on graphite anodes.

The XRD patterns of the regenerated anodes after DI‐water/AA treatments are presented in Figure S5, indicating that the washing process had little influence on the graphite crystal structure since there was very little difference between the washed and pretreated samples.

**FIGURE 3:**
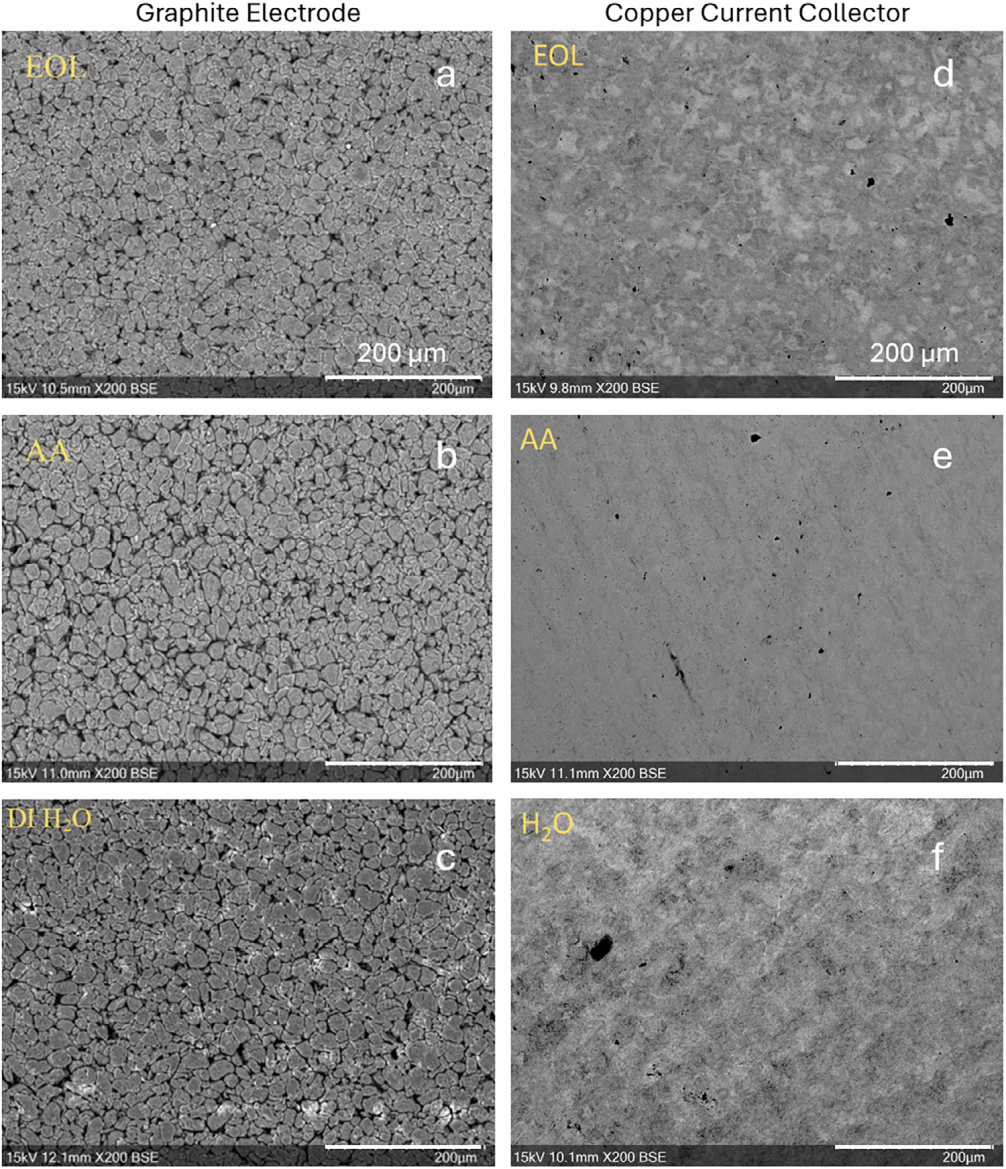
SEM images show 2D microstructures of the anode (1st column) and Cu current collector (2nd column) before and after washing by deionized water and ascorbic acid (AA): (a,d) end‐of‐life sample; (b,d) ascorbic acid treated EoL sample; (c,f) DI‐water treated EoL sample. The scale bars represent 200 µm for all SEM images.

The effects of green washing agents on EoL microstructure are demonstrated in Figure 3. It should be noted that the bright spots in the EoL H_2_O image are instrumental artefacts and not sample contaminations or defects. SEM images (1st column) show a clear increase in inter particle spacing (‘gaps’) after the EoL graphite was washed with ascorbic acid (AA, see Figure 3b) and DI‐water (Figure 3c). The particle spacing in EoL and EoL H_2_O anodes quantified by 3D tomography (Figure 3) will be discussed in detail below. The increased gaps could be attributed to chemical–mechanical factors. Bubbles were visually observed when EoL electrodes were submerged and washed in both water and AA solvents. These could be produced by the reaction of water with unoxidized lithium as follows:

2Li+2H2O→2LiOH+H2

Bubble formation and oscillation might act as a stirring force to remove the surface and inner residues (stemming from side reaction) and widen pre‐existing gaps due to aging. It was also found that the intensity of the bubbles was related to the strength of the acids (pH value); in preliminary research, strong acids (i.e., HCl, HNO_3,_ and H_2_SO_4_) produced the most bubbles, and weaker acids the least.

Table 3 shows that AA offered a better cleaning effect than DI‐water. The concentration of almost all elements decreased after the treatment, except carbon and copper. The increase in observed Cu content is likely due to reaction with AA, exposing the copper foil. There was a significant decrease in the oxygen level for EoL graphite after treatments, from 41.17% to 4.47 (AA) and 8.88% (DI‐water), providing strong evidence for the effectiveness of the green rejuvenation technique. The EDS suggests that the AA‐washed anode had a lower O content than the water‐washed case, as AA is more effective in removing metal oxides. This is also verified by the previous mass measurements (Figure S9). The pH value of AA is lower than DI‐water, facilitating the acid–base reaction. In particular, AA is efficient in removing manganese (which comes from the blended cathode consisting of LMO and NCA [43]).

**TABLE 3:** The elemental composition (EDS) of EoL graphite after washing treatments.

Graphite on electrode						
Element	Atomic (%)	Std	Atomic (%)	Std	Atomic (%)	Std
	EoL		Ascorbic Acid (AA)		H_2_O	
O	41.17	0.17	4.47	0.06	8.88	1.93
C	53.05	0.08	95.31	0.08	89.94	2.16
Si	0.02	0	0.01	0	0.01	0.01
Al	0.01	0.02	0.08	0	0.09	0.07
Cu	0.01	0	0.08	0.01	0.1	0.06
S	0.07	0.03	0.03	0	0.03	0
Mn	0.46	0.02	0	0	0.71	0.2
P	0.01	0	0	0	0	0.02

A comparison of the EoL Cu foil with solvent‐recycled ones was also investigated (2nd column). From a structural point of view, compared to the EoL Cu foil (Figure 3d), there was a dramatic improvement in the surface quality of the Cu foil after washing with AA (see Figure 3e). The SEM shows that a green organic acid can help remove the oxides and impurities on the surface of Cu foil, which could improve the contact, flatness, and electrical conductivity between the anode materials and the foil. The black spots observed on all current collectors are a result of small graphite pieces remaining on the Cu foils. The elemental results obtained through EDS taken from three Cu current collectors are listed in Table 4. It is widely accepted that the main aging mechanism for Cu current collectors is oxidation [44]; evaluation of the oxygen atomic % for all Cu foils, therefore, could inform the treatment required. The first comparison of the EoL Cu foil to the H_2_O‐washed Cu showed a decrease from 17.38 at. % to 5 at.%. There was a further decrease in O to 2.62 at. % for AA‐washed Cu foil, showing that ascorbic acid has a stronger oxide removal capability than DI‐water. The above results as a case study show that efforts on recycling current collectors [45] should not be neglected, as this could be part of a ‘closed‐loop’ to ensure that recyclers both reduce waste and maximize profit recovery, given the value of Cu metal.

**TABLE 4:** The elemental composition (EDS) of EoL Cu current collectors after washing treatments.

Cu current collector						
Element	Atomic (%)	Std	Atomic (%)	Std	Atomic (%)	Std
	EoL		Ascorbic Acid (AA)		H_2_O	
C	37.47	1.15	43.38	1.77	37.30	1.84
O	17.38	0.83	2.62	0.33	5.28	0.18
Si	0.05	0.01	0	0	0.01	0.01
Cu	44.71	2.08	52.36	1.27	55.64	1.73
Mn	0	0	0	0	0	0
S	0.07	0.05	0.18	0.16	0	0

### 3.3 3D Visualization and Quantification of Pretreated and Water‐Washed Anodes

To further improve our understanding of the water‐assisted washing technique without damaging electrode system, high‐resolution X‐ray tomography was used to characterize the morphologies of key samples in 3D.

**FIGURE 4:**
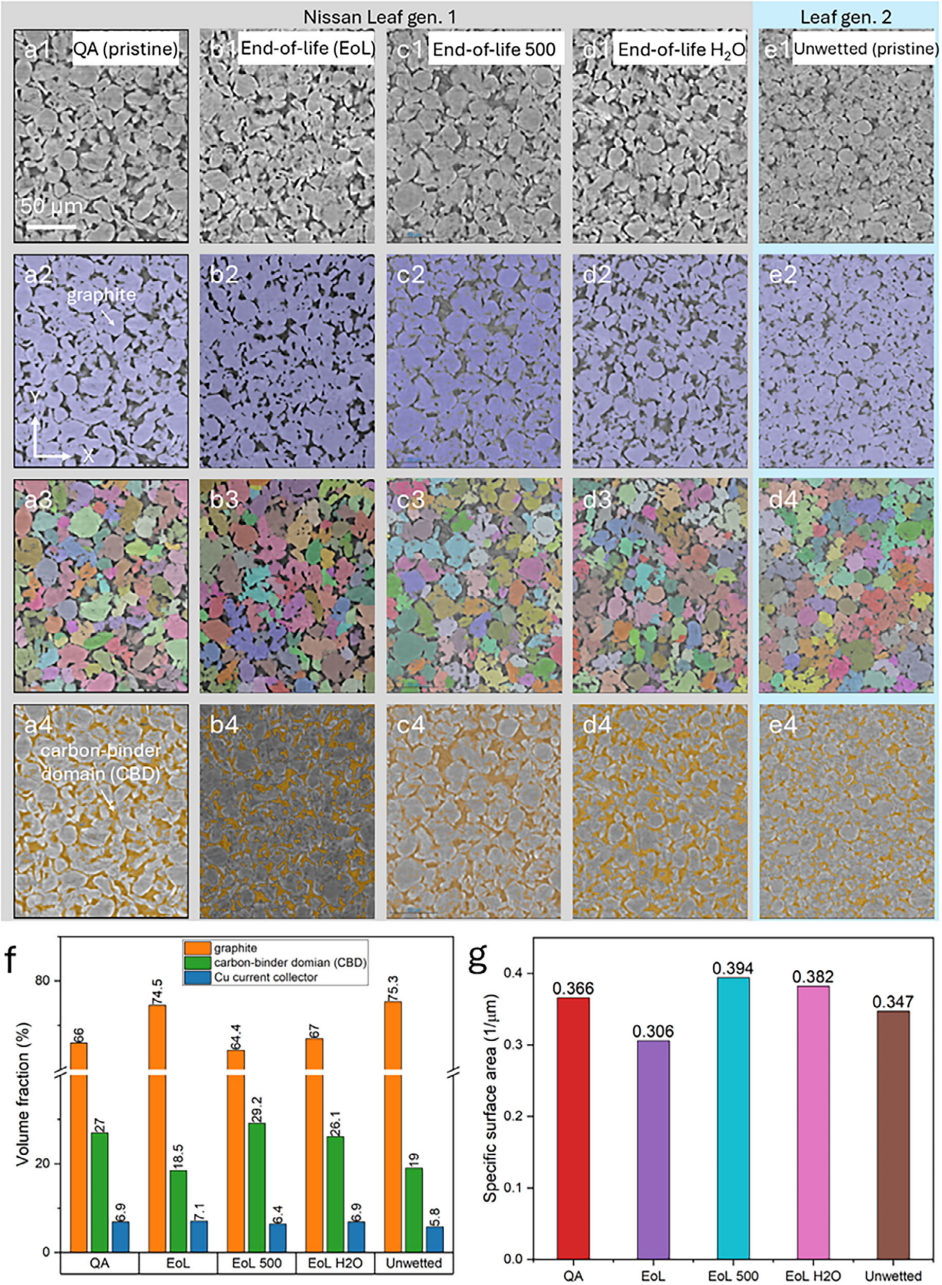
X‐ray µCT images of graphite anodes showing 2D cross‐sectional microstructures under various lifespan conditions and their metrics, including (a1–a4) QA reject (pristine state); (b1–b4) cycled anode at end‐of‐life (EoL); (c1–c4) anodes with an extra 500 cycles after EoL; (d1–d4) water‐washed anode after EoL; (e1–e5) unwetted sample (pristine state); volume fraction (f) and specific surface area (g) for associated samples. AI‐assisted segmented images of graphite (2nd row) and carbon‐binder domain (CBD, 4th row) are presented. Labeled images (3rd row) were obtained through watershed‐based segmentation.

In Figure 4, 3D tomographic images of different anode specimens are demonstrated to complement the SEM observations and X‐ray 2D virtual slices. A smaller region of interest (ROI) of 165 × 445 × 545 pixels (ca. 69 µm × 187 µm × 229 µm) was cropped from the entire volume to better visualize the morphological differences. The reconstructed 2D raw slices (in the middle of the volume) are presented in the first row to show graphite particles (light grey) and carbon‐binder domain (dark black) due to their attenuation differences. Correspondingly, the AI‐assisted segmentation for graphite particles (assigned in purple) and carbon‐binder domain (CBD, assigned in orange) are shown in the 2nd and 4th rows, respectively. Each graphite particle is labeled with an individual color to its neighbors in the 3rd row for further quantification.

In comparison of the two pristine samples, the unwetted gen. 2 (Figure 4e2) exhibited a visibly smaller graphite particle size than the gen. 1 (Figure 4a2), and the distance between graphite particles was generally closer for gen. 2 (Figure 4e4), suggesting a faster ion‐transportation for gen. 2 owing to its shorter route. The gen. 2 exhibited a higher active material loading than the gen. 1 (Figure 4f), with a volume fraction of 75.3%. In addition, gen. 2 graphite had higher sphericity and higher structural homogeneity than the gen. 1 samples in terms of size, shape, and distribution. The previous cycling and EIS results (Figure 1) confirm the structural observation here. This is also an example that demonstrates how better material morphology and electrode manufacturing drive improved performance.

The size of graphite particles appeared to gradually increase as a function of their lifetime, from first‐life (Figure 4a2) to end‐of‐life (EoL, see Figure 4b2) to second‐life (EoL 500, see Figure 4c2). Again, this could be due to side reactions at the interface where the amorphous surface coating forms a layer that thickens in use and when exposed to air [39]. Furthermore, the anode expansion caused a negative effect on its neighboring CBD, as the connected channels were compressed that led to many isolated channels at EoL (Figure 4b4). After second‐life use (EoL 500), the CBD phase became more inhomogeneous as shown in Figure 4c4. The effect of water‐assisted washing on morphology is presented in the 4th column to compare against the pristine state (1st column). As expected, the size of graphite particles decreased after the DI‐water washing (Figure 4d2), with the gap between graphite becoming more prominent than in other cases. The result indicates that DI‐water to some extent can remove the aging‐induced layer covering the graphite, which helps recover the performance during regeneration. Indeed, the morphological metrics (Figure 4f,g) for recovered anodes were very similar to those of the pristine sample in terms of volume fraction and specific surface area. It should be noted that some defective graphite particles are still present after washing, as washing cannot repair lattice‐damaged [46]. particles.

**FIGURE 5:**
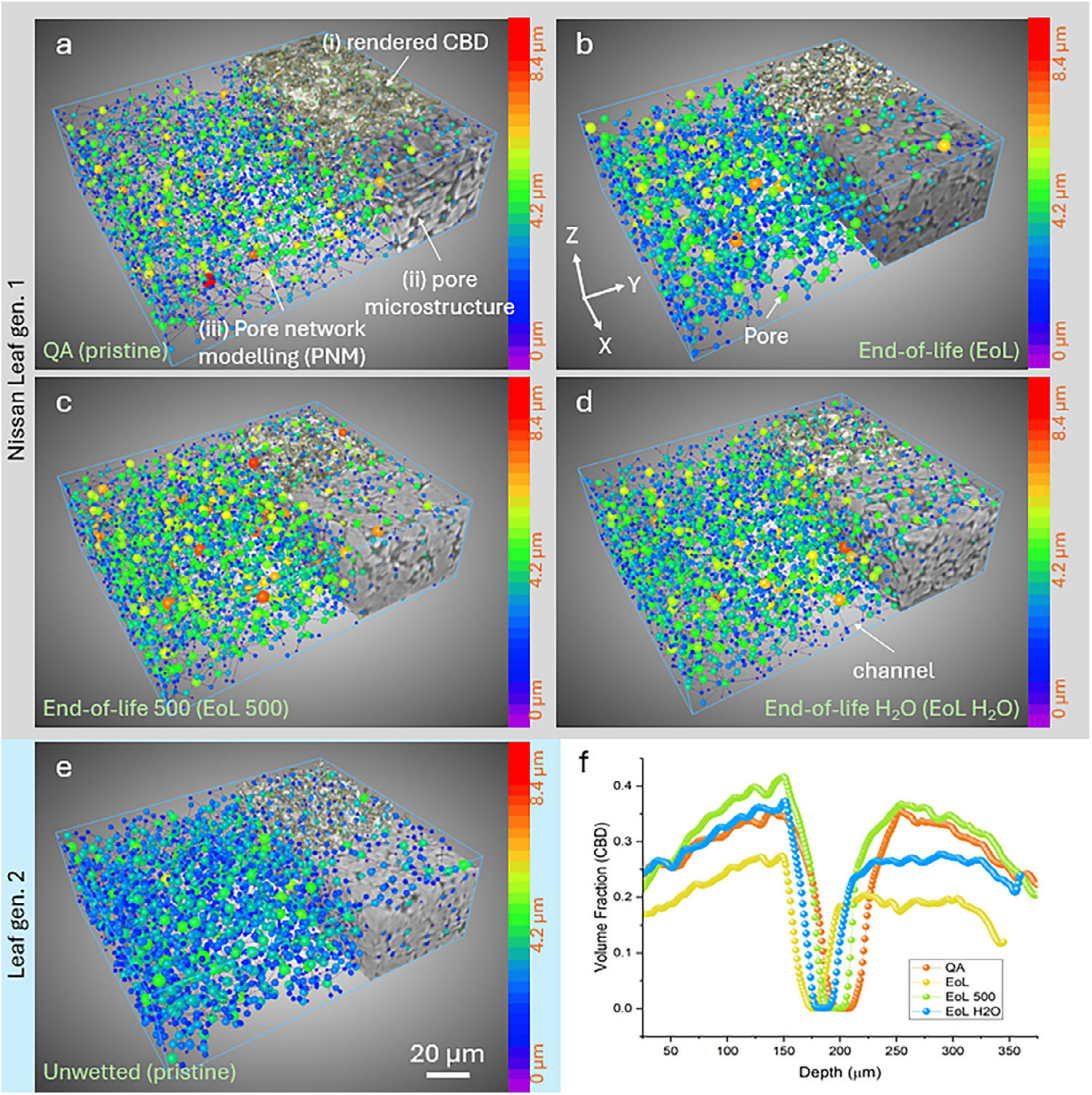
The pore networks modeling (PNM) of graphite anodes and their pore distributions. They are (a) QA reject (pristine state); (b) cycled anode at EoL state; (c) anode with an additional 500 cycles after EoL; (d) water‐washed anode after EoL; (e) unwetted sample (pristine state). (f) the volume fraction of porosity (CBD phase) along the depth (Z‐axis) direction. Note: The CBD phase is represented as a series of spheres connected by narrower throats/channels (shown as lines) through PNM. The assigned‐color spheres represent pores in the anode scaled according to their equivalent diameter (in the range of 0–8.4 µm). The scale bar represents 20 µm for all graphs.

To study the structure beneath the surface, an approach called pore network modeling (PNM) is performed on the porous CBD phase (binary images) to understand the structural degradation against lifetime and the effect of CBD and porosities on the ion transportation. 3D visualizations of pores and their channels, microstructure, and the rendered CBD are superimposed to present in Figure 5. The bottom of the samples (along with the Z direction) faces the Cu current collector (not shown here). The Z‐axis represents the direction from current collector to the separator, while the *X–Y* plane corresponds to the cross‐section of the anode. Our PNM results have high accuracy, thanks to high‐resolution imaging and subsequent AI‐assisted segmentation; the latter offers a definitive segmentation of CBD phase that minimizes errors and bias. The spheres and cylinders represent pores and throats/channels, respectively, and were automatically assigned various colors to reflect and represent the diameter and channel length.

It is obvious that the gen. 2 electrode (Figure 5e) exhibited a very uniform size distribution of micropores compared to the gen. 1 electrodes (Figure 5a), with sizes ranging from ∼2 (assigned blue) to ∼4 µm (assigned green). This is further evidence that a homogeneous structure (CBD) leads to better performance, as the travel pathway of ions would be more uniform. The homogeneity in pore size emphasizes how highly optimized industrial anode coating techniques are compared to their academic lab equivalents.

The second‐life anode (EoL 500, see Figure 5c) had the greatest variation in pore size, with several large spheres (labeled in red and orange) mixed with the medium‐sized (labeled in green) and small spheres distributed within the volume. The extreme deviations in microstructure, coupled with their poor electrochemical performance confirm that spent anodes in late life would require further recycling treatment as the highly inhomogeneous morphology may restrict both ion and electrolyte transportation within the electrode structure.

DI‐water washing was found to improve the homogeneity of the CBD (see Figure 5d). It is thought that soaking and washing the aged anode in the DI‐water may clear the blockage of the internal channels, thus recovering the functionality of the CBD, at least in part. However, it is unclear if the washing process might flush away parts of the original CBD phase, leaving those regions completely disconnected, with no paths available for ion transportation. This might be applied to those regions away from the current collector, where they are exposed more to the washing agent. Moreover, the morphology between the electrode and the current collector shows minimal variation before and after washing. It was found that the local volume fraction of CBD phase (Figure 5f) was greater than those for overall measurements (Figure 4f). Nevertheless, more in situ efforts will be needed to establish a quantitative relationship between the performance of regenerated anodes and proposed rejuvenation processes.

**FIGURE 6:**
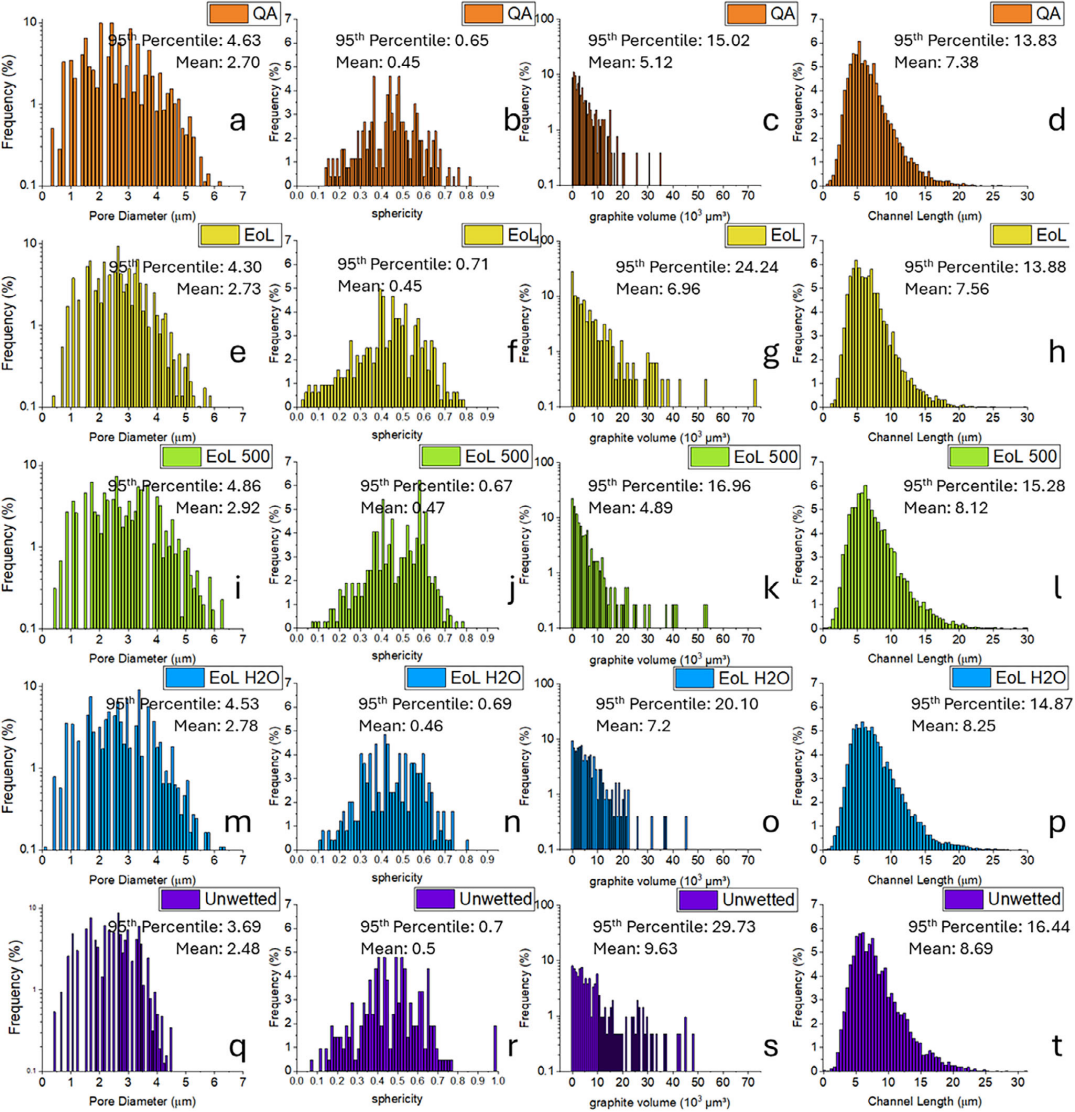
Quantitative data for graphite anode samples under different conditions, including (a–d) QA reject (pristine state); (e–h) end‐of‐life (EoL) state; (i–l) extra 500 cycles after end‐of‐life; (m–p) DI water‐washed state after end‐of‐life; (q–t) unwetted sample (pristine state, gen. 2). The particle distributions of pore diameter (1st column) and their sphericity (2nd column), the volume distributions of graphite particles (3rd column), and the length distribution of pore throat/channel network (4th column) for associated samples.

In Figure 6, the quantitative PNM results illustrate a gradual increase in the average pore diameter (1st column) over the lifetime. The gen. 2 anode (Figure 6q) had the smallest pore diameters, with a mean value of 2.48 µm. As for the gen. 1 anodes, the diameter increased from 2.7 µm in the pristine state to 2.73 µm (EoL state), and further to 2.92 µm at the second‐life (EoL 500) case. The pore diameter is believed to affect the electrochemical performance; an increase in pore space increases the likelihood of electronic isolation of the graphite particles, which will increase the internal resistance resulting in a lower battery capacity.

Despite the growth trend and the pore size (for the gen. 1 anodes) aligning well with results measured by mercury intrusion porosimetry (MIP, see Figure S7), the pore diameter for the gen. 2 case measured by the MIP was smaller than that measured by PNM. This discrepancy may arise from the fact that MIP is a destructive method that relies on the Washburn model to estimate pore size, and very few materials fulfil the ideal requirements of the model.

The analysis also revealed a gradual increase in channel length (4th column of Figure 6) with sample aging, starting at an average of 7.38 µm in pristine (Figure 6d) and extending to 8.12 µm in end‐of‐second‐life state (Figure 6l). The increase in channel length indicates that ion pathways became longer as the anode aged, which necessitates more time for ion transport in aged samples compared to fresh ones. Our case demonstrates that X‐ray µCT can offer rich information beneath the sample surface, significantly improving understanding of degradation and regeneration processes.

Quantitative data for different graphite samples are also presented in Figure 6. The distribution of sphericity confirms tomographic observations: first, the gen. 2 graphite had the highest sphericity value of all samples, suggesting the graphite was more spherical rather than irregular; second, there was a minor difference regarding the mean value of sphericity for the gen. 1 anodes. The difference probably indicates that gen. 1 graphite was of synthetic origin whereas gen. 2 was of natural origin, and together the results suggest that the shape of graphite was not the main factor governing the performance after regeneration.

The appearance of a particularly low sphericity zone (0–0.1, on the left side of the X‐axis) in the EoL electrode (Figure 6f) may be attributed to particle fracture. These fragments often have an irregular shape and low sphericity. It is observed that particle distribution histograms broaden with aging, which can be attributed to particle fractures alongside larger agglomerates breaking up, owing to weakening of the PVDF binder.

**FIGURE 7:**
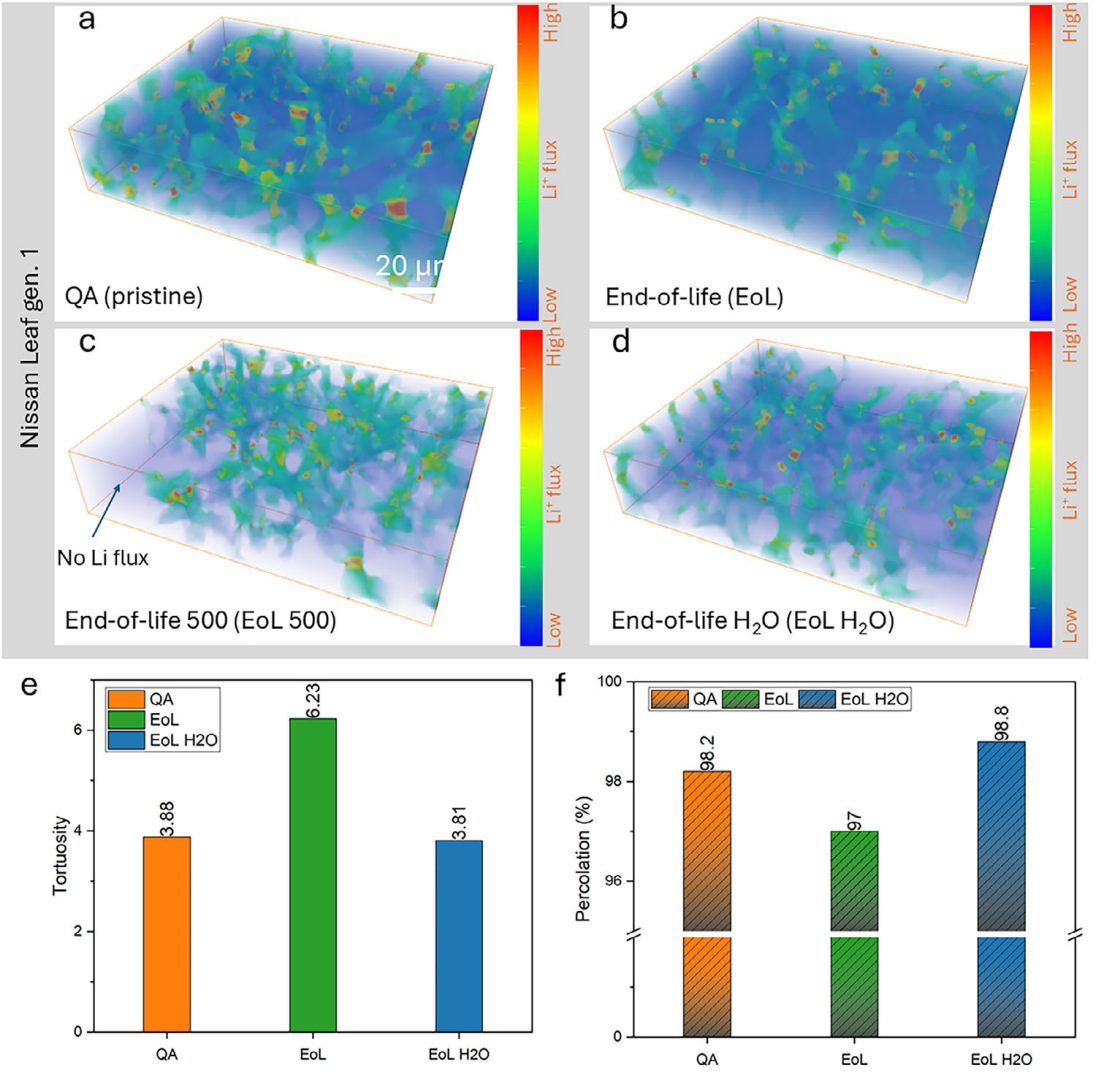
3D visualization of simulated Li^+^ ion fluxes of the porous CBD phase against various lifetime stages: (a) pristine state (QA reject); (b) EoL state; (c) EoL 500 state; (d) deionized water‐washed EoL state. Quantitative data for the tortuosity factors (e) and percolation (f) along the Z direction.

Image‐based simulation was performed on the same sub‐volumes along the Z‐axis direction (electrode to current collector) through the Taufactor in Matlab to understand further the effect of CBD microstructure on the cell performance for the gen. 1 anodes. 3D flux simulations are shown in Figure 7, along with quantitative data for associated tortuosity and percolation.

In Figure 7a, the flux channels with considerable size are homogeneously connected to each other and distributed within the domain, enhancing the ion conductivity. In contrast, the density of flux decreased for the EoL anode (Figure 7b), restricting diffusion and related ion transportation after the first life. Again, this was a result of the amorphous layer causing the blockage. The generation of many thinner channels after the second life (EoL 500, see Figure 7c) may be linked to further fracture of the agglomerates. 3D visualization of the EoL and EoL 500 electrodes shows that flux density was much lower than that in the pristine case, with considerable flux channels disappearing in some regions, which could be the main reason for degraded performance. It seems clear that water washing helped restore performance by reopening flux channels (Figure 7d). In comparison to the pristine and EoL H_2_O anodes, DI‐water washing did not reverse the CBD environment to its original condition, however; instead it only restored a portion of the initial flux.

The quantitative data on tortuosity (σ) in Figure 7e corroborate the 3D visualization. The value of σ_EoL H2O_ (3.81) was very close to that of σ_*p*ristine_ (3.88), but they were both smaller than σ_EoL_ (6.23). A higher value of σ often implies an increased resistance or inefficiency in transport processes. Therefore, the results indicate that ions may have to travel longer in the EoL anode than in the pristine and regenerated cases. It should be noted that tortuosity measured by X‐ray CT is more effective than that measured by MIP. This is mainly because we can quantify the specific direction (e.g., Z‐axis) rather than relying on an overall metric.

Figure 7f exhibits the reverse trend for the percolation data; the EoL anode had a smaller percentage of percolation (97%) than the pristine (98.2%) and regenerated (98.8%) electrodes. As percolation describes the flow through interconnected pathways influenced by the connectivity of porous electrodes in this study [47], the low percolation of the EoL electrode suggests reduced connectivity compared to other cases, again indicating that the water‐assisted washing can restore the connectivity in EoL anode materials. This approach offers a pathway for evaluating degradation and optimizing recycling routes by visualizing the channels and networks directly.

### 3.4 Performance of Rejuvenated Electrodes

**FIGURE 8:**
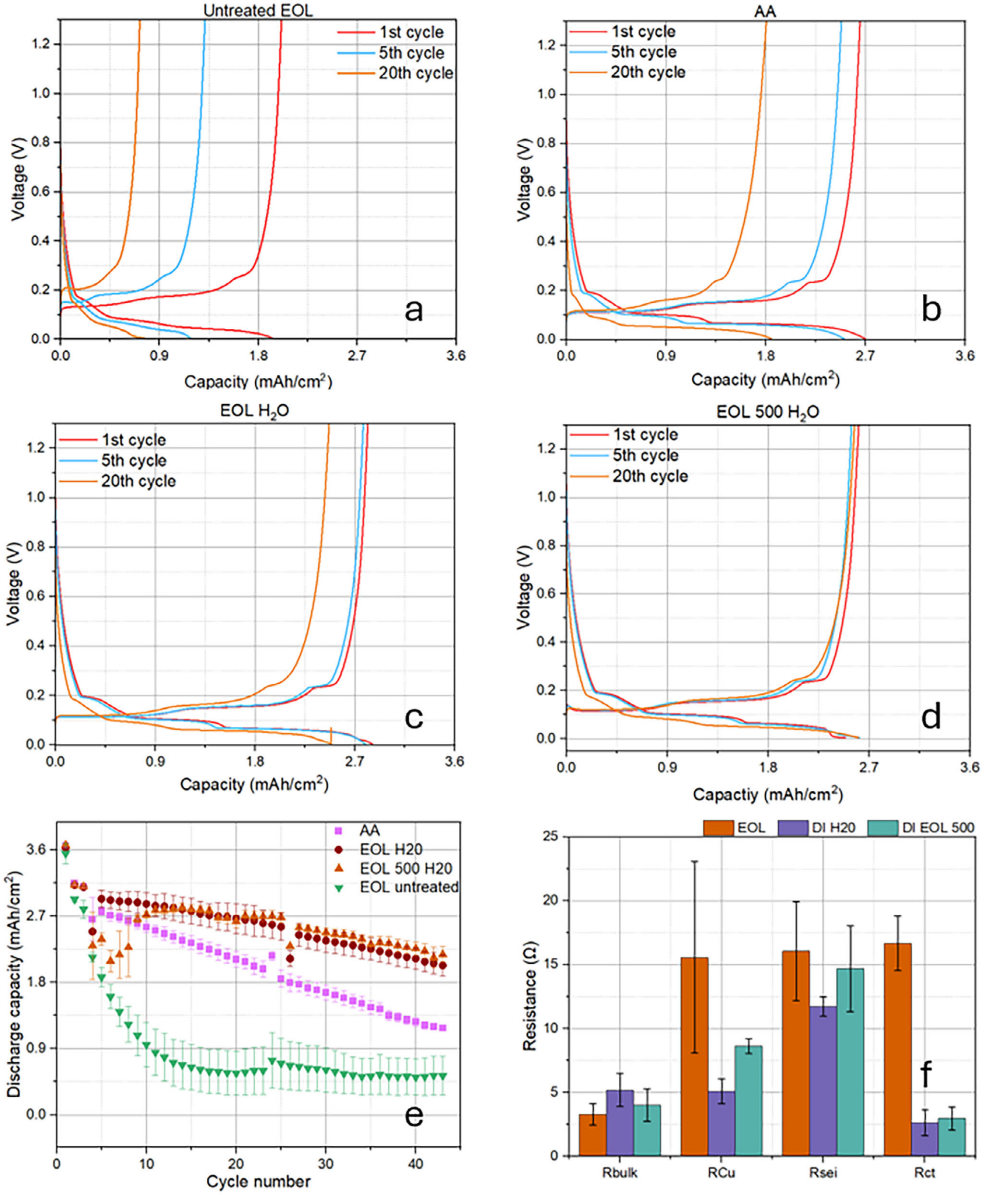
Electrochemical performance and EIS measurements for regenerated and upcycled graphite anodes. The voltage‐capacity profiles after the 1st, 5th, 20th cycles for (a) end‐of‐life sample, (b) ascorbic acid treated on EoL sample, (c) deionized water treated EoL sample, (d) deionized water treated EoL 500 sample; (e) the discharge capacity as a function of cycle number for the associated four samples, with the error bars representing the standard deviation; (f) EIS extracted parameters for water‐washed anodes showing *R*_bulk_, *R*_Cu_, *R*_SEI_ and *R*_CT_ taken after the formation cycle.

To evaluate the performance of the rejuvenated electrodes, the EoL and EoL 500 anodes were washed (either water or AA), assembled into a coin cell and cycled. Figure 8a–d shows the 1st, 5th, and 20th full cycles extracted from the main cycling phase at C/10. The two main features of interest on these graphs are the discharge plateaus (which indicate the extent of lithiation) and the point at which the charge and discharge lines intercept (which indicates the degree of polarization). For the formation cycles, no cell polarization was observed as the cycling rate used in the formation protocol was exceptionally slow.

The recycled graphite showed a typical discharge profile (similar to pristine graphite) in Figure 8 and Figure S10. The small bump at 0.7 V is typical for the first lithiation step that corresponds to the reduction of the electrolyte at the graphite–electrolyte interface. The reduction of the electrolyte subsequently forms the SEI layer that coats the surface of the electrode and shields the electrolyte from further reduction by the graphite, meaning this peak is only present on the first formation cycle. The irreversible depletion of the electrolyte means the first cycle capacity figure is always artificially higher than the true reversible capacity. The subsequent plateaus within the *E*_𝑐𝑒𝑙𝑙_ vs capacity graph indicate various lithiation stages within the graphite. According to the Daumas–Herold model, the intercalation process can be broken down into 5 stages (see Figure S10); the L indicates that the lithium ions are not perfectly ordered during the transition. The first stage of the intercalation process labeled 1L is described as a random organization of lithium ions (similar to a liquid) within the graphite lattice resembling a solid solution. The transition from stage 4 to stage 1 represents the gradual filling of the space between the graphene layers going from every 4th layer (C_42_Li), to every 3rd layer (C_36_Li), to every 2nd layer (C_12_Li), then finally every layer (C_6_Li), hence the label of stages 4 through to 1. Upon complete lithiation of the graphite a phase change occurs from the ABABAB (hexagonal) and/or ABCABCABC (rhombohedral) phases to a hexagonal AAAA stacking phase. (De)lithiation mechanisms proposed in pristine graphite are expected to apply to recycled graphite as the structure remains the same after washing.

The EoL anode (Figure 8a) as a control sample had the worst performance of all electrodes, showing i) a lack of extended plateaus on the discharge step indicating limited lithium‐rich (C_12_Li, C_6_Li) phase formation within the graphite structure, and ii) increased cell polarization with increasing cycle number. The water‐washed electrodes (Figure 8c,d) had better capacity retention than the AA‐washed ones (Figure 8b), showing that fewer impurities do not necessarily equate with better performance in washed anodes. Surprisingly, after washing, the EoL 500 demonstrated the best electrochemical performance of all with good columbic efficiency and low polarization. This implies that the DI‐water helped restore (de)intercalation efficiency after washing. This result also implies that it is entirely possible to recover the graphite after second‐life applications that extend battery life and still maintain a flow of recovered graphite material suitable for reuse in new batteries at eventual end‐of‐life.

The performance of regenerated anodes is summarized in Figure 8e. Correspondingly, the specific capacity was presented in Figure S11. The measured areal capacity from water‐washed anodes was exceptional, showing excellent capacity retention as a function of cycle number after 40 cycles. This indicates that the reduced performance of these EoL graphite anodes was largely not due to any degradation of the graphite material, which remained fundamentally sound and could be recycled directly without the use of aggressive chemicals. Comparing the water‐washed electrodes (red and orange) to the AA‐washed one (pink), there was a significant difference in discharge capacity of 1 mAh/cm^2^ after 40 cycles.

To better understand the causes of polarization and differences in performance, associated EIS measurements were taken after formation to evaluate the degradation mechanisms. The resistances extracted from the EIS fits for the water‐washed samples are shown in Figure 8f.

The reduction in the Cu contact interface resistance (*R*_Cu_) is attributed to the removal of the non‐conducting surface coatings and impurities. It was found that the *R*_Cu_ value for the EoL 500 water‐washed electrode was higher than that for the EoL water‐washed electrode, which could be attributed to continuous degradation of the Cu itself (repeated cycling causing oxidation that reduces the electrical conductivity at the interface). Also, a reduction in *R*_SEI_ for both EoL and EoL 500 water‐washed electrodes was observed compared to the unwashed EoL case, showing that the water washing technique was effective in removing surface impurities and residues. The larger SEI resistance seen in the EoL 500 sample may be due to increased edge plane exposure, caused by particle fragmentation from volume changes during repeated cycling. The most significant reduction among all measured parameters was the reduction in the charge transfer resistances (*R*_CT_) for the EoL and EoL 500 samples after washing, which can be attributed to the removal of the built‐up SEI layer. This reduction helps shorten the ion diffusion length for (de)intercalation as well as reducing the resistance of the process.

**FIGURE 9:**
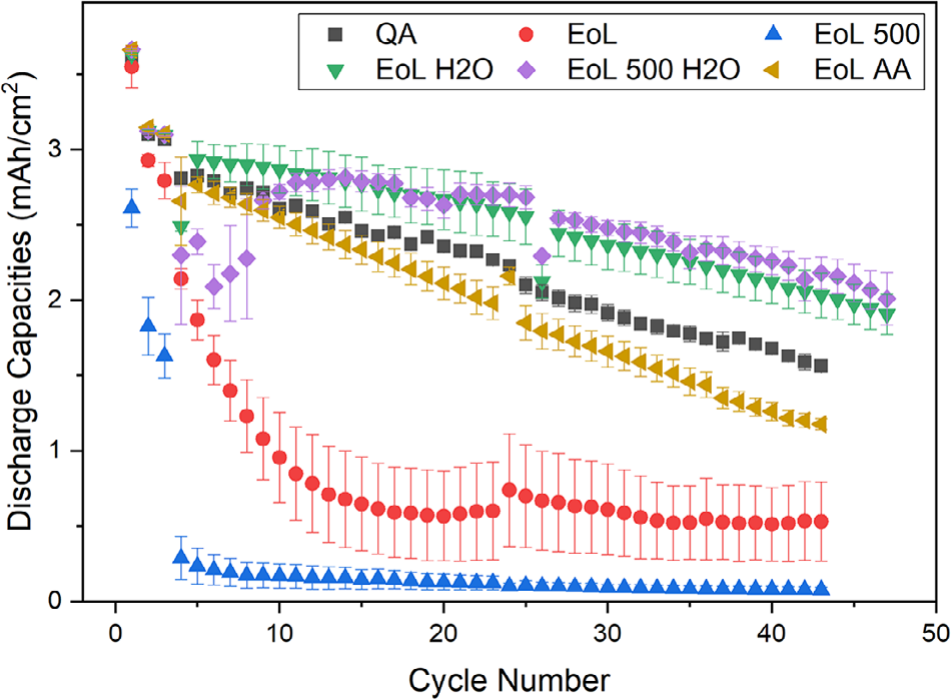
Electrochemical performance measurements for the unused (QA), end‐of‐life (EoL), end‐of‐second‐life (EoL 500), regenerated graphite anodes.

To better understand the effectiveness of the washing processes, electrochemical performance of all anodes is plotted in Figure 9. Results demonstrate that all regenerated anodes had much superior performance than those scrapped anodes. They also show that the DI‐water‐washed anode outperformed both the unused (2.36 mAh/cm^2^) and end‐of‐life (0.56 mAh/cm^2^) anodes after 20 cycles, highlighting the efficiency of this straightforward regeneration technique for these graphite materials.

## 4 Conclusion

For the first time, advanced characterization techniques, including X‐ray tomography, SEM, EDS, XRD, Raman, and mercury intrusion, along with electrochemical performance and EIS measurements, have been used to investigate systematically pristine (from a commercial manufacturing plant), spent (from a retired EV), second‐life, and rejuvenated anodes. Benchmark tests were performed using conventional tools to narrow down the best samples for detailed X‐ray tomography. Green solvents, including DI‐water and ascorbic acid, were selected as environmentally friendly washing agents to recover spent battery anode system after first life and second life. This approach is a potential alternative to lengthy hydrometallurgy processes and avoids the use of inorganic acids that usually generate hazardous wastewater.

The effectiveness of green‐solvent‐assisted washing was demonstrated by comparing the masses, chemistries, structures, and morphologies of the associated anodes. Our results show that good electrochemical performance can be achieved using regenerated graphite anodes washed with DI‐water (for fabricating new Li‐ion batteries), with a stable discharge capacity of ∼2.65 and ∼2.21 mAh/cm^2^ over 20 and 40 charge–discharge cycles at 0.1 C, respectively.

A high‐resolution X‐ray imaging technique was employed to obtain the 3D microstructure for selected anodes. 3D visualization and quantification of the morphology of electrode system have been demonstrated to provide a pathway toward a deeper understanding of the structural changes beneath the electrode surface after water treatments. Our results indicate that DI‐water removes impurities accumulated in pores and channels of used anodes, which helps restore their vitality. This is clearly evidenced by pore/channel distribution analysis and flux simulations, including metrics such as tortuosity. This unique proof‐of‐concept case study illustrates the potential of advanced characterization to generate new insights into battery anode recycling and regeneration, bridging missing links between green recycling processes, regeneration performance, and material structure.

In summary, this work demonstrates that it is entirely possible to regenerate spent graphite anodes, even after second‐life applications, for circulation in new batteries with comparable performance to similar anodes regenerated after end‐of‐first‐life, and this could be a viable route to extend battery life. Our study shows that, even after extended real‐world use, graphite anodes can be directly rejuvenated in a facile manner without removal from the current collector, implying that the graphite remains fundamentally sound and direct recycling with minimal environmental impact should be possible. Although washing agents can remove surficial contamination coated on graphite particles, they cannot repair graphite fragmentation or structural fracturing within the graphite unless high‐temperature heat treatments are applied. Thus, the long‐term effects of such defects in rejuvenated electrodes need to be considered in more detail in future work.

## Author Contributions

L.S., conception, formulation, investigation and methodology (excluding X‐ray tomography), validation, data curation, formal analysis, interpretation, writing, and reviewing and editing; A.T.S., formal analysis, interpretation, and reviewing and editing; Y.D., formal analysis and interpretation; S.Z., data curation; J.C., data curation; F.I., investigation; R.J., supervision and resource; P.K.A., supervision and reviewing; P.R.Sl., supervision, reviewing and funding acquisition; C.M., supervision, reviewing and resource; P.A.A., project administration, supervision, reviewing and editing the final manuscript, resource and funding acquisition; P.R.Sh., project administration, supervision, reviewing and editing the final manuscript, resource, and funding acquisition; W.D., project administration, conception, investigation and methodology, validation, data curation, formal analysis, interpretation, writing‐combining‐completing original draft, reviewing and editing the final manuscript.

## Conflicts of Interest

The authors declare no conflicts of interest.

## Supporting information




**Supporting File**: smll72362‐sup‐0001‐SuppMat.docx.

## Data Availability

The data that support the findings of this study are available from the corresponding author upon reasonable request.
