# Supplementary material for: Advanced Characterization and Rejuvenation of End‐Of‐Life Lithium‐Ion Anodes: Toward the Development of a Green Upcycling Route
Source: Small. 2026 Jan 20;22(14):e12626. doi: 10.1002/smll.202512626 (PMC12965121; doi:10.1002/smll.202512626)
Supplement: Supplementary file 1 — Supporting File: smll72362‐sup‐0001‐SuppMat.docx. [file SMLL-22-e12626-s001.docx]

**Supplementary Materials**

**Advanced characterization and rejuvenation of end-of-life lithium-ion anodes: towards the development of a green upcycling route**

by Luke Sweeney et al.

Corresponding Authors: Wenjia Du ([wenjia.du@eng.ox.ac.uk](mailto:wenjia.du@eng.ox.ac.uk))

**
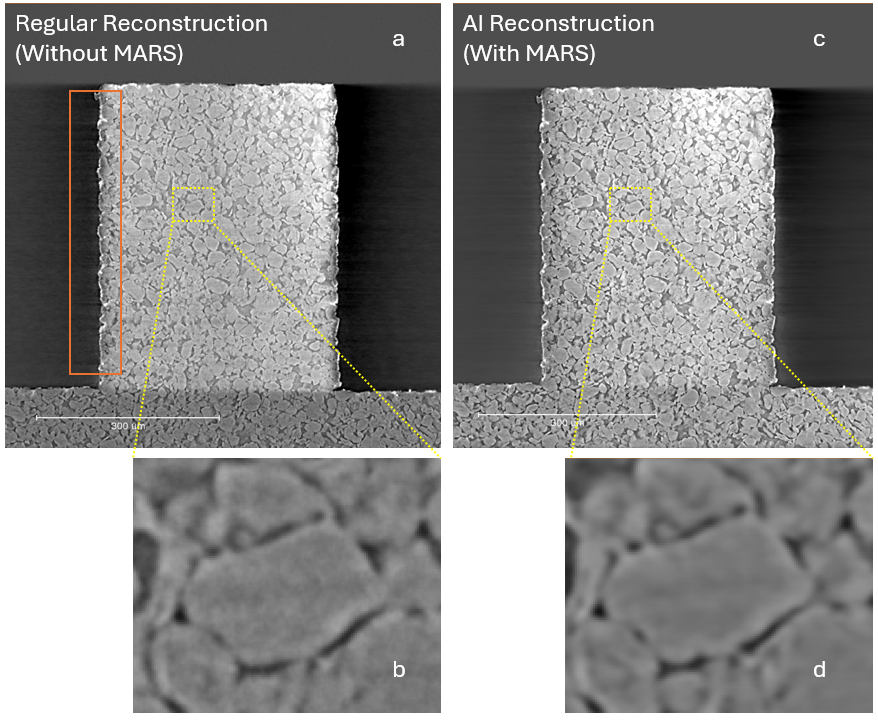
**

**Figure S1. The reconstructed CT images of QA anodes without (a-b) and with (c-d) artificial intelligence (MARS by Zeiss)**. The zoom images (2^nd^ row) show the difference in signal-to-noise ratio and image contrast at feature edge.

**
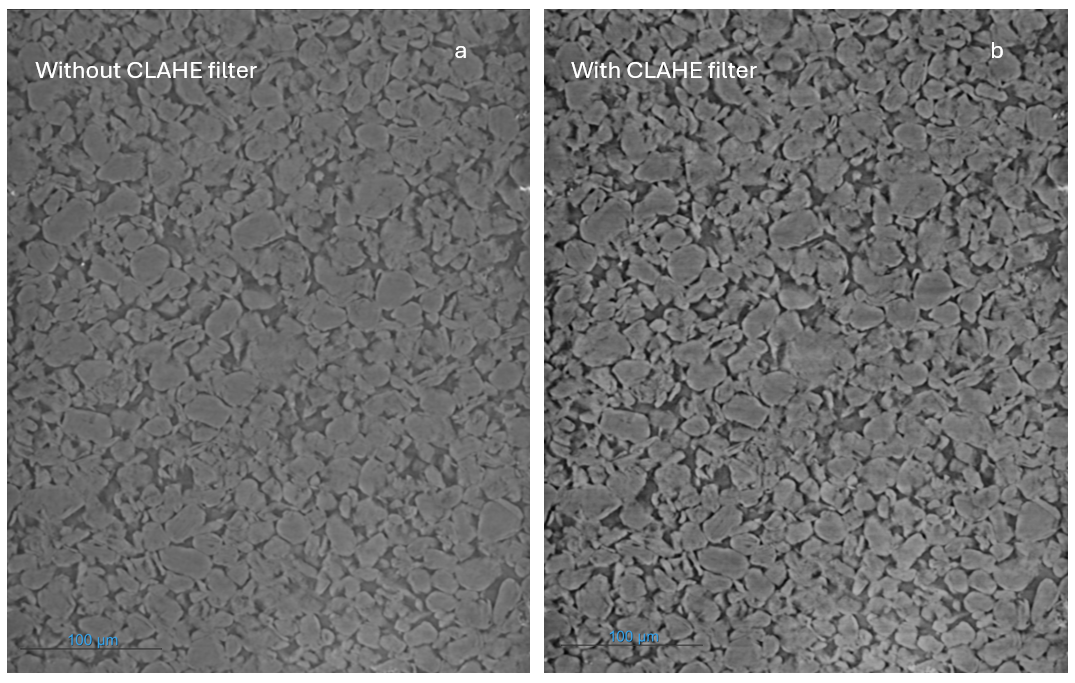
**

**Figure S2. 2D images of QA anodes without (a) and with (b) the application of a CLAHE filter** after AI-assisted (MARS) reconstruction to further enhance edge contrast, facilitating CBD analysis.

**
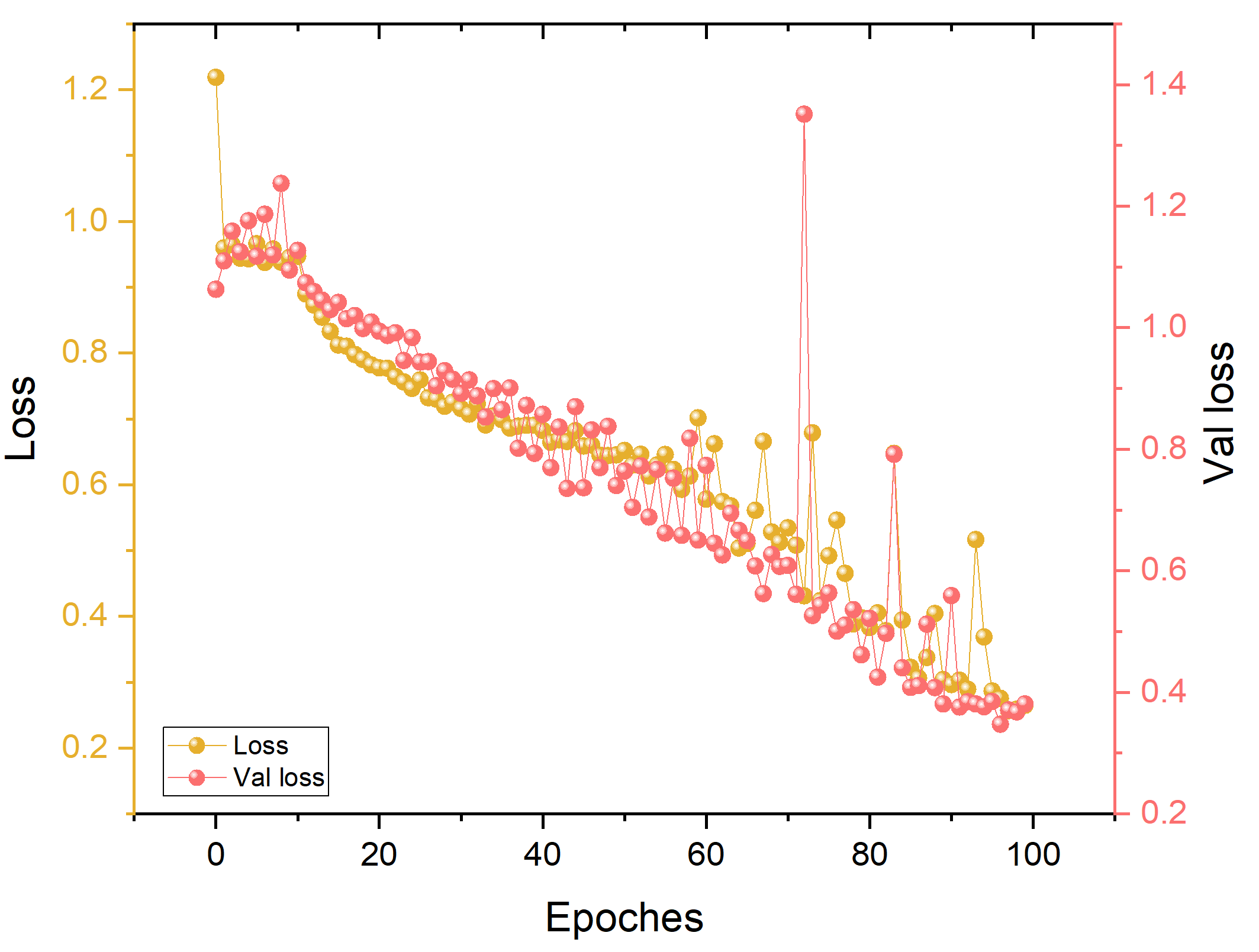
**

**Figure S3. The training profile of U-net showing the loss function (red) against the epochs.**

**
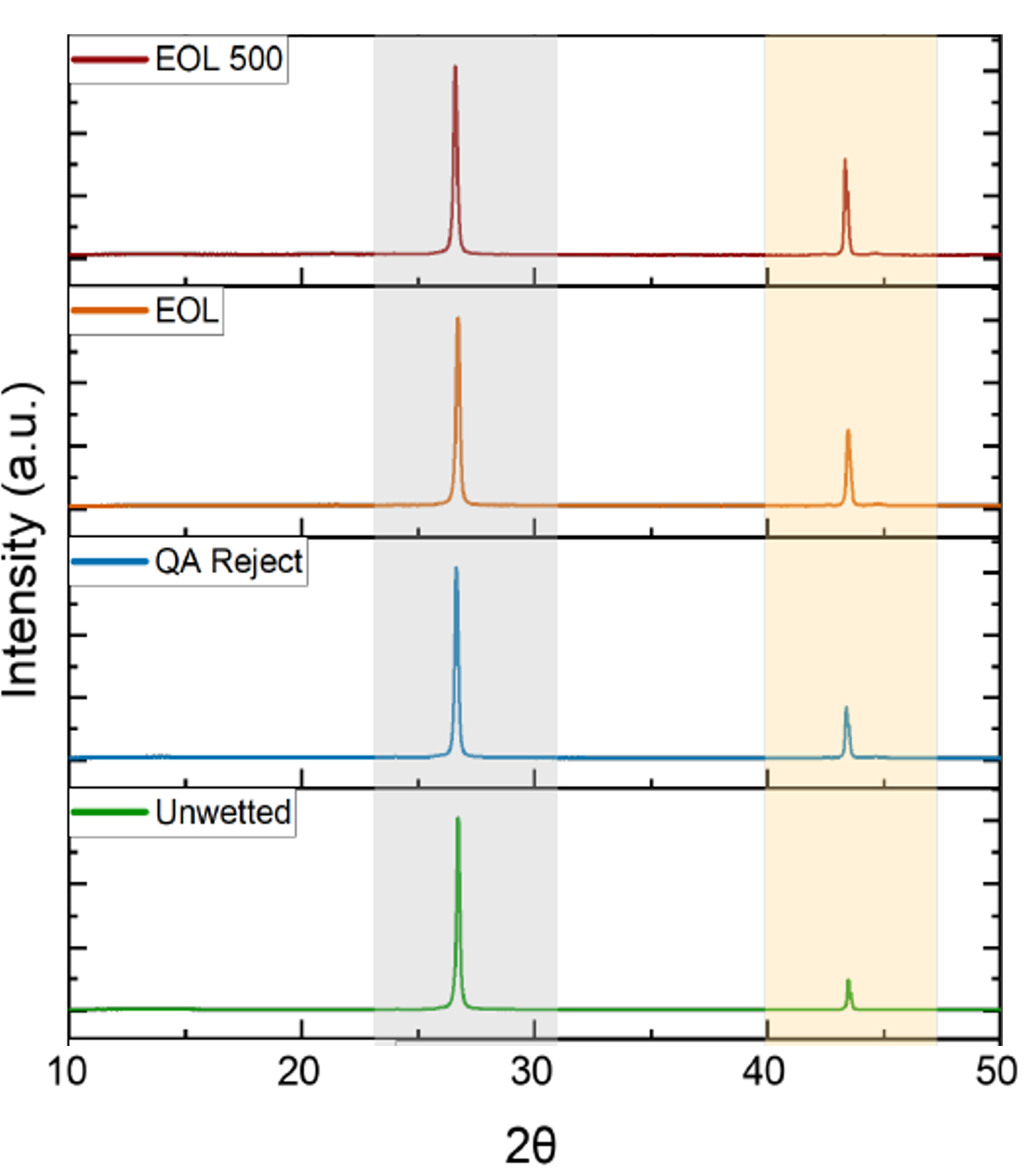
**

**Figure S4. XRD patterns of the anodes in pristine and EoL states, highlighting the most prominent graphite peak (002) within the grey section and the copper peak (111) within the orange section.**

**
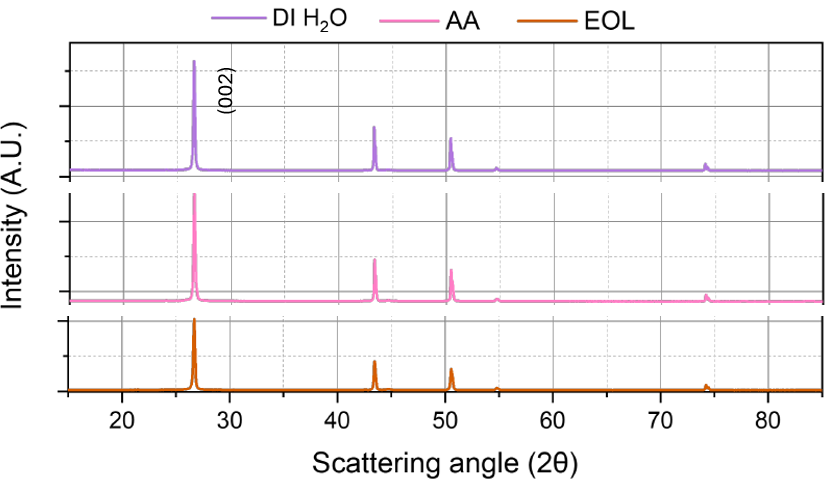
**

**Figure S5. XRD patterns of the pretreated (bottom), ascorbic acid-washed (middle) and DI-water (top) washed anodes for comparison.**


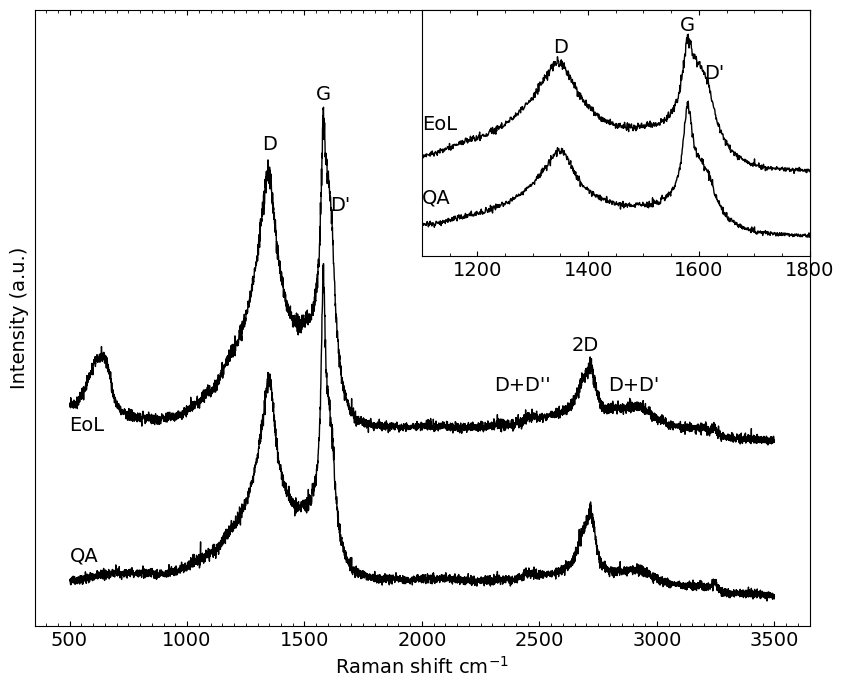


**Figure S6. Ex-situ Raman spectroscopy of QA and EOL anodes.**

**
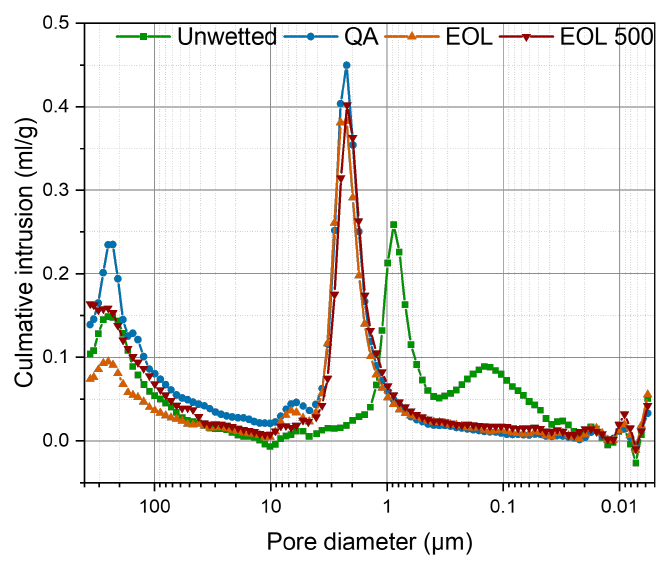
**

**Figure S7. Culminative mercury intrusion plot showing the pore size distribution of the different electrode structures.** It should be noted that this plot is from destructive measurements to compare with those measured by X-ray tomography and PNM simulation.

**
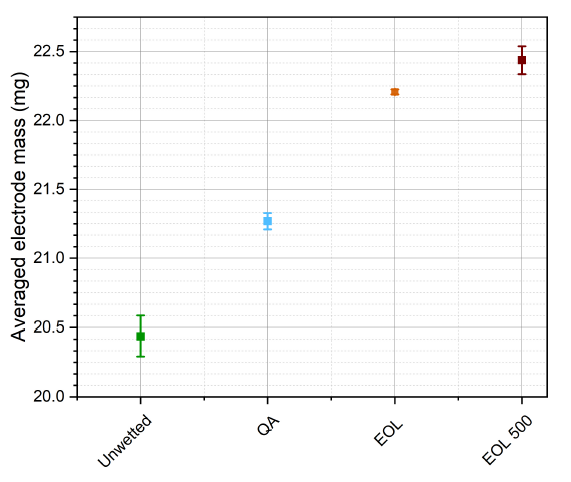
**

**Figure S8. The weight of pre-treated electrodes (taken from punched 1.2 cm diameter disks) measured by using a micro balance with the standard deviations shown by the error bars.**

**
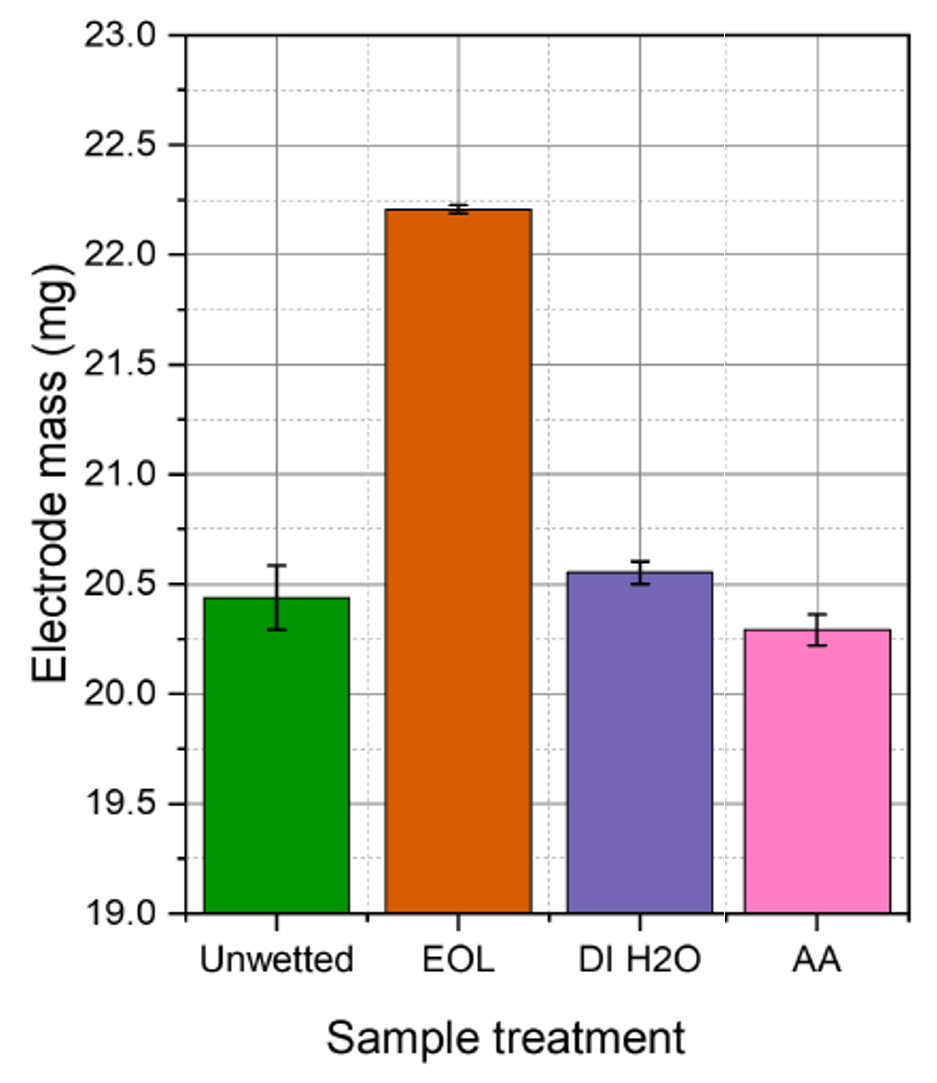
**

**Figure S9. Comparison of the electrode masses taken before electrochemical testing showing the change in overall electrode mass after different washing techniques.**

**
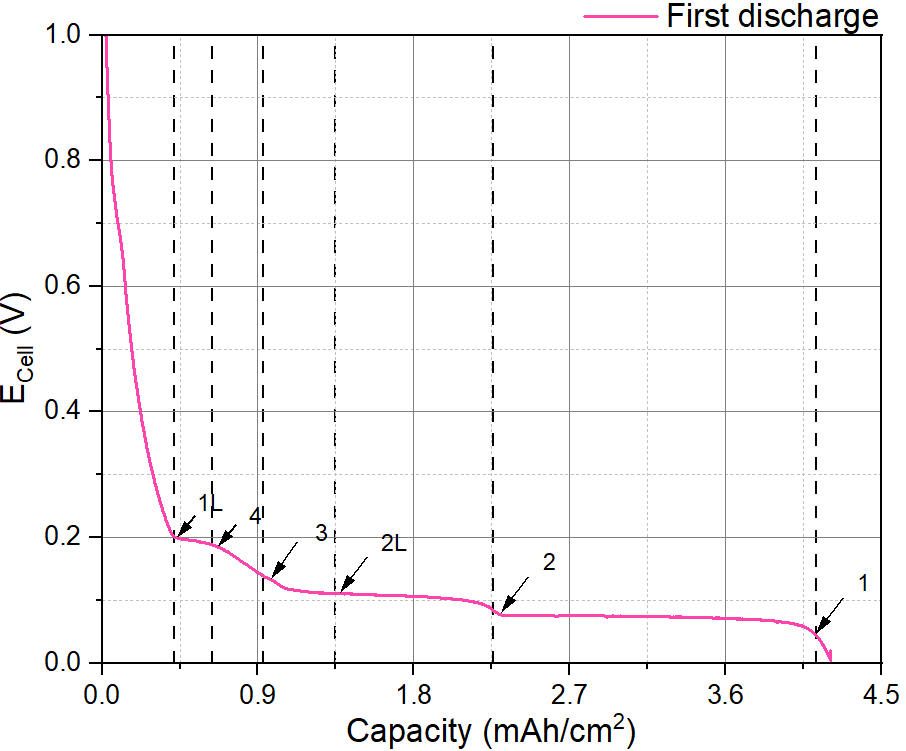
**

**Figure S10. An example of the SEI formation in the first discharge cycle of a graphite lithium metal half-cell. The data are from an unwetted electrode manufactured by Nissan.** Stage 1L (Dilute stage): Li ions start to enter the graphite, Stage 4: every fourth graphene layer is occupied by Li ions, Stage 3: Li ions occupy every third layer, Stage 2L: Li is approaching every second layer but still disordered, Stage 2: Li ions are well-ordered in every second layer, Stage 1: Li occupies every graphene layer.


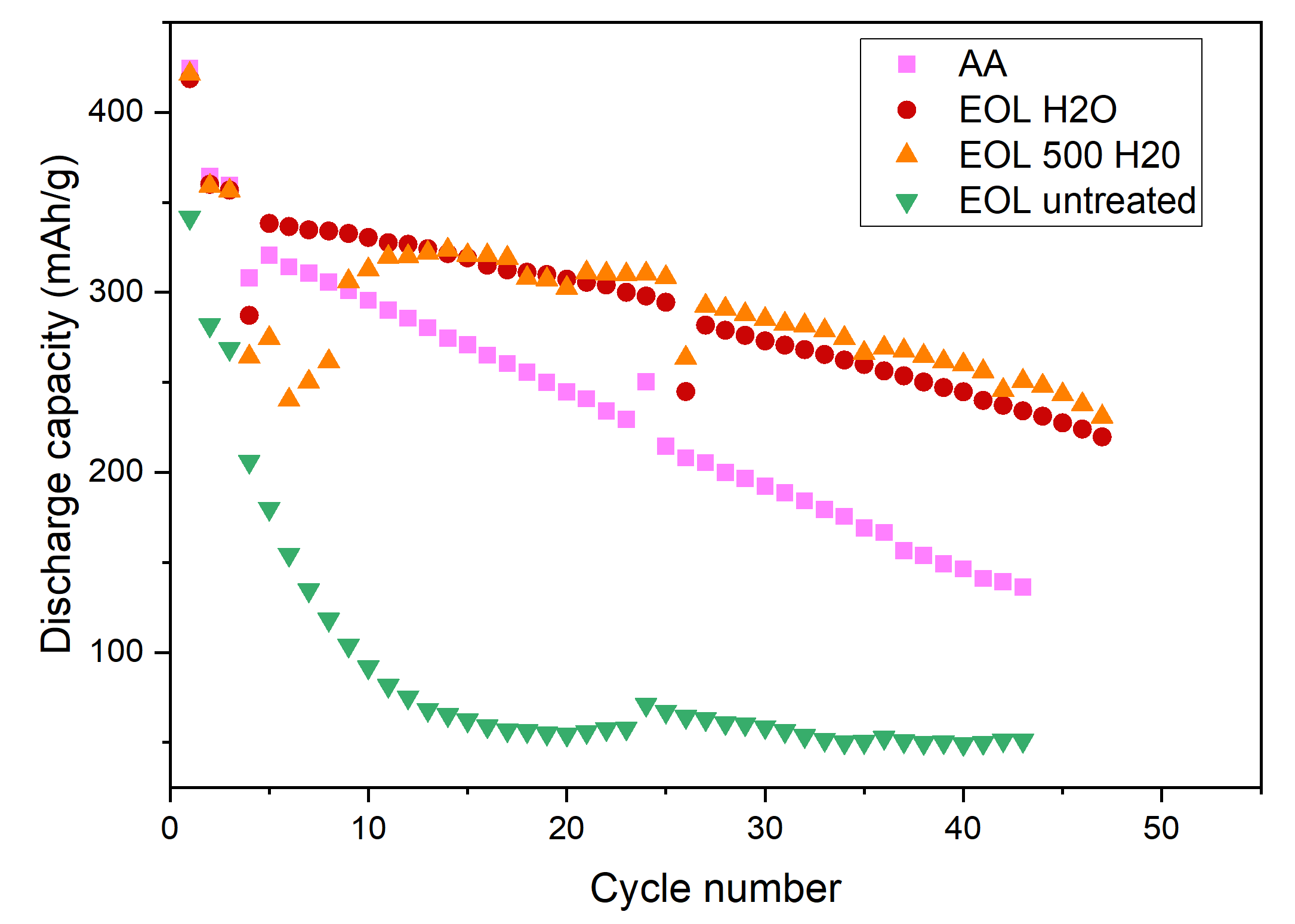


**Figure S11. The specific discharge capacity as a function of cycle number for the associated four samples.**
